# Beta band stability over time correlates with Parkinsonian rigidity and bradykinesia

**DOI:** 10.1016/j.expneurol.2012.04.024

**Published:** 2012-08

**Authors:** S. Little, A. Pogosyan, A.A. Kuhn, P. Brown

**Affiliations:** a Department of Clinical Neurology, Oxford University, 6th Floor, West wing, John Radcliffe Hospital, Headley Way, Oxford, OX3 9DU, UK; b Sobell Department of Motor Neuroscience and Movement Disorders, UCL Institute of Neurology, Queen Square House, Queen Square, London WC1N 3BG, UK; c Department of Neurology, Charité, University Medicine Berlin, Campus Virchow Klinikum, Augustenburger Platz 1, 13353 Berlin, Germany

**Keywords:** CV, Coefficient of variation, DBS, Deep brain stimulation, KS, Kolmogorov–Smirnov test, LFP, Local field potential, LZC, Lempel–Ziv complexity, STN, Subthalamic nucleus, UPDRS, Unified Parkinson's disease rating scale, Parkinson's disease, Deep brain stimulation, Beta, Oscillations, Biomarker

## Abstract

Abnormal oscillatory activity in the basal ganglia is increasingly implicated in the pathophysiology of Parkinson's disease. Such activity is recorded in patients in the form of oscillations in the local field potential (LFP) picked up in the subthalamic nucleus. Previous studies have focused on correlations between features of the time averaged power or amplitude spectrum of the LFP and the clinical state, either off medication or in response to levodopa. However, average spectral densities do not take account of time variant spectral properties and we hypothesised that these dynamic properties of the spectrum of the LFP would contain additional information about clinical state. Here we assess the variability in LFP amplitude over time using the coefficient of variation (CV), evaluating this with regard to clinical state off medication and in response to levodopa in two datasets. The CV of activity in the high beta frequency band was found to be correlated with clinical state off levodopa (rho = − 0.59, p < 0.001) and this was shown to be complementary, rather than redundant, to spectral amplitude in a multiple regression analysis, selective for rigidity–bradykinesia and highly focal. Similarly, a strong correlation was found between change in clinical scores and change in high beta CV following levodopa (rho = − 0.66, p = 0.004). This too was selective for rigidity–bradykinesia and non-redundant to spectral power in a multiple regression model. Our results indicate that temporal stability in the beta band is correlated with rigidity–bradykinesia. It is suggested that loss of beta reactivity is deleterious to basal ganglia function over and above any concomitant change in absolute level of beta synchrony. The CV of LFP beta band amplitude may potentially provide an additional index of clinical state suitable for feedback control in closed loop stimulation therapy.

## Highlights

► Variability in beta band amplitude correlates with rigidity–bradykinesia in Parkinson's. ► Correlations occur with motor impairment off medication. ► Correlations also occur with change in motor impairment upon treatment. ► Correlations are frequency and symptom specific, as well as spatially focal.

## Introduction

There is an increase in correlative evidence suggesting that exaggerated oscillatory synchronisation in the beta frequency band may be related to motor impairment in patients with Parkinson's Disease (Jenkinson and Brown, 2011). Such synchronisation is usually indirectly measured as the average spectral amplitude or power of beta frequency band activity in the LFP recorded in basal ganglia sites targeted during functional neurosurgery. LFP fluctuations largely reflect the temporal and spatial summation of presynaptic neuronal activity (Goldberg et al., 2004; Magill et al., 2004; Mitzdorf, 1985) and therefore LFP amplitude or power indexes the strength of synchronisation, the density and (up to an electrode dependent limit) the spatial extent of the involved neural pool. However, a further variable is often overlooked, and this is the constancy of these features over time. Synchronisation and the size of the affected neural pool can change from moment to moment, leading to fluctuations in spectral amplitude or power over time that affect the average signal. Consider, for example, two networks. One is almost completely synchronous or very extensive for a brief instant, and then barely synchronous or very small for the rest of the time. Another network is modestly synchronised and extensive throughout. The two networks may lead to similar time averaged LFP power but the impact upon normal functioning may potentially be very different. We have previously reported that the spatial extent of beta band phase synchronisation in the region of the STN correlates with rigidity — bradykinesia (Pogosyan et al., 2010). We now test whether the temporal stability of synchronisation as inferred from LFP amplitude also correlates with rigidity — bradykinesia.

A correlation between the temporal stability of beta band amplitude and motor impairment would be important for several reasons. First, fluctuations in amplitude might imply the existence of processes that can disrupt pathological synchrony, which might provide clues for new therapeutic approaches, as in epilepsy (Loddenkemper et al., 2001). Secondly, the temporal stability of beta band amplitude might provide a faithful indicator of current motor state suitable as a feedback signal in the closed loop control of therapeutic deep brain stimulation (Rosin et al., 2011). Temporal stability might provide a faithful feedback signal in its own right, or, if proven to carry non-redundant information, through combination with other measures.

We elected to assess the temporal stability of the amplitude of the LFP in the beta range with a simple measure, the standard deviation of the time varying beta band amplitude normalised by the mean beta band amplitude, otherwise known as the coefficient of variation (CV). There is some hope that the CV of LFP amplitude might correlate with clinical state, given the correlation of a complexity measure of beta activity with motor impairment (Chen et al., 2010). However, the CV and complexity, as estimated using the Lempel–Ziv complexity are not equivalent. The Lempel–Ziv complexity captures not only linear dependencies in the structure of the LFP over time but also nonlinear interactions between frequencies (Chen et al., 2010) making it a more difficult statistic to estimate and a less intuitive one. We also elected to assess the strength of correlations between motor impairment and CV in the lower and upper beta frequency band, and to investigate this both with respect to the off medication state and change with medication, — aspects not explored with the Lempel–Ziv complexity. Consideration of the lower and upper beta frequency bands was motivated by studies which suggest a differential functional and pathological significance between these frequency ranges (López-Azcárate et al., 2010; Marceglia et al., 2006, 2007; Priori et al., 2004).

## Methods

### Patients and surgery

We investigated the relationship between time related fluctuations in the amplitude of the beta band signal and motor impairment, as indexed by the motor UPDRS, in the off medication state at rest and in response to levodopa treatment in two independent archival datasets; an intraoperative off medication rest dataset and a post-operative dataset which included data from on and off medication. The surgical target was the STN and the DBS electrode used was model 3389 (Medtronic Neurological Division, Minneapolis, USA).

#### Rest off medication dataset

Eighteen patients with advanced idiopathic PD were recorded intra-operatively in the off medication state (mean age 60.4 ± (SEM) 7.0 yrs, disease duration 15.2 ± 5.4 yrs, mean pre-operative UPDRS motor score 42.9 ± 15.7 off drugs and 11.1 ± 7.4 after levodopa challenge). These patients have been reported previously (Pogosyan et al., 2010); however, the earlier study only investigated the correlation between LFP phase coherence and clinical features. LFPs were recorded from bipolar pairs of adjacent electrode contacts to maximise spatial selectivity. This afforded a series of three bipoles, 01, 12 and 23, from the four electrode contacts of each electrode, where contact pair 01 was the most caudal. Intra-operative recordings involved a staged descent in 2 mm steps from above the STN with periods of recording at each level. All patients showed a step increase in beta power between contacts 01 on entering the STN of at least 100%. This depth has been termed the physiologically defined target level (Yoshida et al., 2010). Full details of the patients, surgery, recordings and physiological targeting are included in the previous report (Pogosyan et al., 2010). Data from this cohort were amplified, pass band filtered between 1 and 80 Hz and sampled at 184 Hz (Biopotential Analyzer Diana, St Petersburg, Russia). Thereafter LFPs were examined off-line in Spike2 software (Cambridge Electronics Design, Cambridge, UK).

#### On–Off medication dataset

Ten patients (20 sides) were recorded in the off state and following levodopa challenge in the post-operative period prior to battery and stimulator implantation (age 60.6 ± 2.5 yrs, disease duration 11.9 ± 1.4 yrs, pre-operative UPDRS motor score 45.1 ± 2.5 off drugs and 22.4 ± 4.1 after levodopa challenge). Full details of patients, surgery and recording have previously been reported separately for nine of the subjects (Kühn et al., 2006a) and one subject (case 5 in (Kühn et al., 2006b)). LFPs were recorded from bipolar pairs of adjacent electrode contacts as above. Data from this cohort were amplified and filtered at 1–250 Hz using a custom-made, high impedance amplifier (which had as its front end input stage the INA128 instrumentation amplifier, Texas Instruments Incorporated 12500 TI Boulevard Dallas Texas, USA) and recorded through a 1401 A-D converter (Cambridge Electronic Design) onto a computer using Spike2 software (Cambridge Electronic Design). Signals were sampled at ≥ 625 Hz.

#### Spectral analysis

**Fig. 1:**
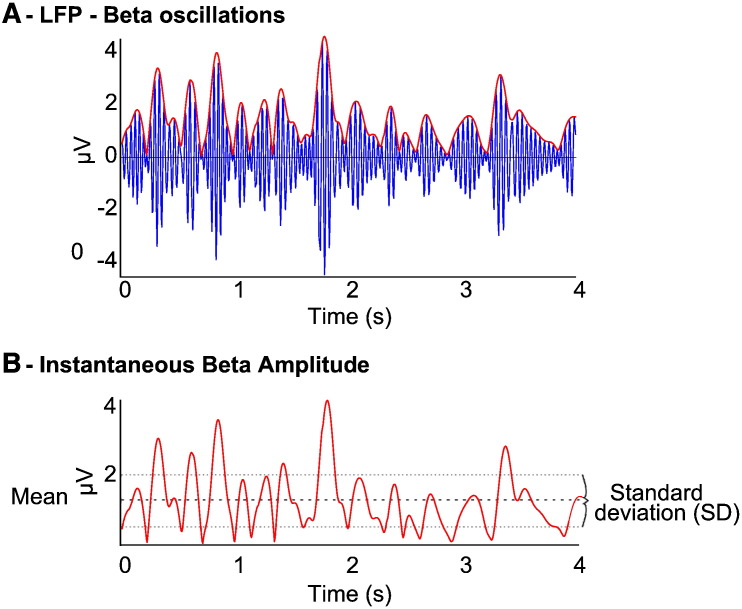
Schematic showing amplitude variability in beta in one subject and corresponding instantaneous amplitude time series. [A] Filtered beta LFP signal from subject 12 (intraoperative rest data) demonstrating marked fluctuations in beta amplitude over time and a low hemibody UPDRS score = 23. [B] Instantaneous amplitude time series with mean amplitude (thick dashed line) and standard deviation also illustrated (thin dashed lines). Instantaneous amplitude is calculated by convolution of the LFP with an appropriately scaled/frequency specific wavelet to determine the amplitude of the selected frequency band at all points in time. The standard deviation of the instantaneous amplitude time series captures the variability of amplitude over time for that frequency. This is then normalised by the mean amplitude in that frequency band to derive a scale invariant measure of amplitude variability — the coefficient of variation of amplitude over time (CV), for each record.

Spectral decomposition with a continuous wavelet transform was performed for both datasets using a Morlet wavelet, due to its efficient time frequency resolution and lack of assumptions regarding stationarity (Muthuswamy and Thakor, 1998; Pogosyan et al., 2010). Convolution of each LFP time series for each subject was carried out with an array of appropriately scaled wavelets to generate a complex time series for each frequency band between 1 and 80 Hz for the duration of each recording, the modulus of which denoted the instantaneous amplitudes for each frequency at all points during the recording. The wavelet transformation is in a sense therefore a band-pass filtering of the LFP such that the resulting time series shows the magnitude of oscillations over the time of the recording in the specific filtered frequency (Fig. 1). As can be seen, there was variation in the amplitudes at the specific frequencies that then were used to calculate the coefficient of variation (CV) by dividing the standard deviation of each frequency specific amplitude time series by the corresponding mean amplitude. This afforded a single value of CV for each frequency for the duration of the whole record. As such, for each subject a single value of amplitude variability (CV) was obtained for each frequency resulting in 80 frequency specific CVs (1–80 Hz) per subject for further correlation.

The CV is therefore scale invariant and relatively immune from confounds introduced at the between subject level such as differences in stun effects or small variation in targeting between sides and subjects. Recording lengths were 65 ± 2.4 s (SEM) for the intraoperative dataset and 286 ± 15 s (SEM) for the levodopa response dataset. Processing was performed in MATLAB (v. 7.11.0, R2010b, The Mathworks, Natik, MA, USA) with custom written scripts.

#### Correlations

The CV averaged across the frequencies within the low beta (beta 1 = 12–20 Hz) and high beta (beta 2 = 21–33 Hz) frequency bands for each side was correlated with clinical scores derived from the motor UPDRS across subjects. The clinical scores were total hemibody score (sum of unilateral UPDRS motor score sub-items 20–26), rigidity-bradykinesia hemibody score (sum of unilateral UPDRS motor score sub-items 22–26) and rest tremor hemibody score (sum of unilateral UPDRS motor score sub-items 20 and 21 for arm and leg) contralateral to the recording site. The percentage change of UPDRS for each item was calculated as ([hemibody scoreON − hemibody scoreOFF] / hemibody scoreOFF) × 100. Similarly, percentage changes in the CV was calculated as [CVON − CVOFF] / CVOFF and in the signal amplitude as [AmplitudeON − AmplitudeOFF] / AmplitudeOFF.

Kolmogorov–Smirnov testing revealed that the intra-operative rest data were not normally distributed and therefore correlations were performed using Spearman's correlation coefficient (rho) and data normalised using a Boxcox power transformation (lamda = 0.3) prior to multiple linear regression. The treatment change dataset fitted a normal distribution (KS p > 0.05) and therefore was not transformed prior to multiple regression. Statistical analyses were performed using SPSS version 19 (SPSS Inc., Chicago, IL, USA).

## Results

### Intra-operative rest recordings off medication (18 subjects, 36 sides)

**Fig. 2:**
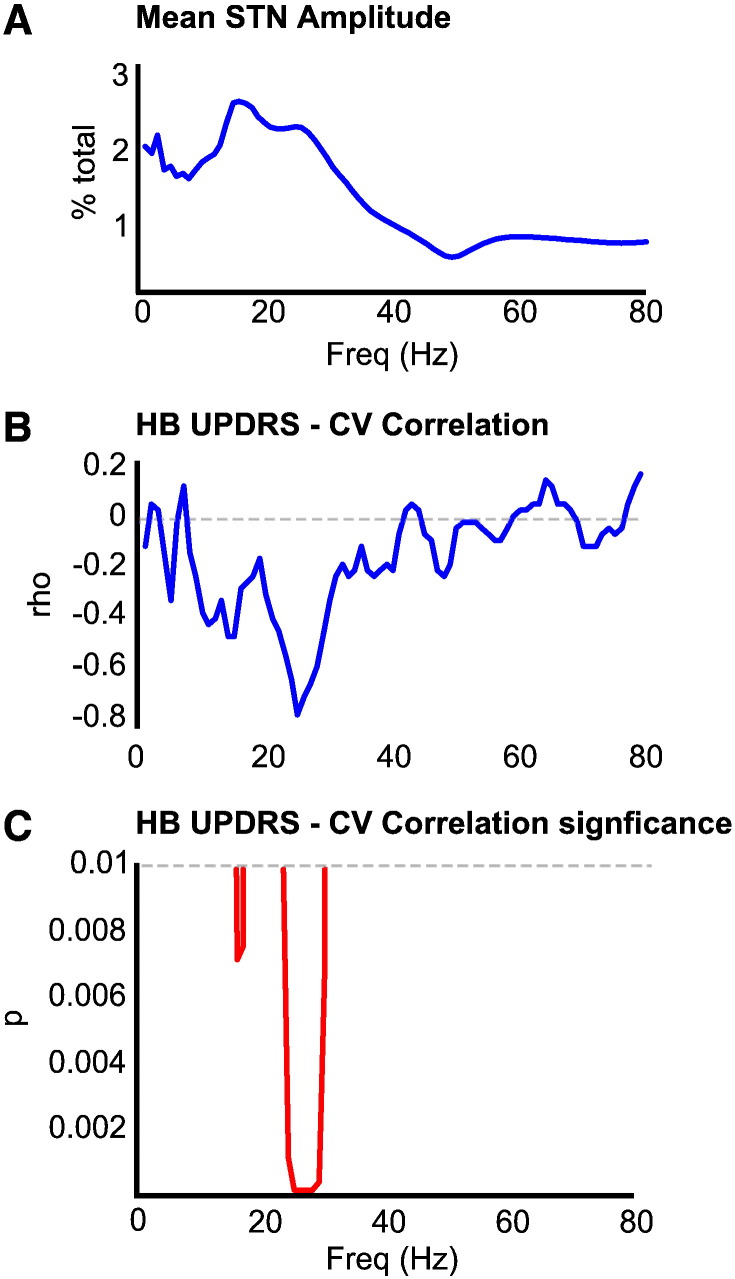
Spectral correlations. [A] Mean STN LFP amplitude spectral density across all subjects, shown as % of total in each 1 Hz frequency band. There are peaks at 16 and 25 Hz, in the beta 1 and beta 2 ranges, respectively. [B] and [C] show Spearman's correlation coefficient (rho) and its significance between the CV of STN LFP amplitude at a given frequency and contralateral hemibody (HB) motor UPDRS scores. There is a strong and broad correlation between the STN LFP CV at a given frequency and contralateral hemibody motor UPDRS scores in the beta 2 range.

Spectral analysis of the LFP recorded at the physiologically defined target level revealed that all 18 subjects had at least one spectral peak within the beta band (13–35 Hz). The power spectrum averaged across all records demonstrated two defined peaks at 16 Hz and 25 Hz, within the low beta (beta 1) and high beta (beta 2) frequency bands, respectively (Fig. 2A).

**Fig. 3:**
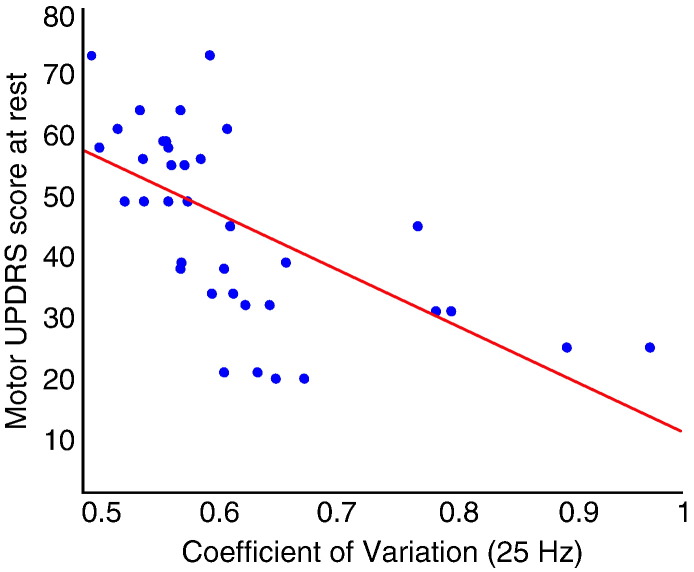
Scatter plot of CV and contralateral hemibody motor UPDRS off medication. Shown for the frequency (25 Hz) of the peak of activity in the Beta 2 range in the group mean spectrum (Fig. 2A). There is a negative correlation between CV and UPDRS off medication (rho = − 0.75, p < 0.001).

The CV of intra-operatively recorded amplitude was calculated with 1 Hz resolution over 1 to 80 Hz and correlated against the presurgical motor UPDRS score. The correlation between the CV of the amplitude envelope and UPDRS scores was significant and broad, albeit restricted to the beta 2 range (Figs. 2B,C). This relationship was strongest at the frequency of the peak (25 Hz) in the beta 2 range of the group mean power spectrum (rho = − 0.75, p < 0.001; Fig. 3). Correlations were negative so that a higher CV was associated with more modest motor impairment in the contralateral limbs.

Having confirmed a correlation between hemibody motor UPDRS scores and the temporal variability of oscillatory activities in the beta frequency band, we separately calculated the mean CV across the frequencies within the beta 1 and beta 2 ranges and correlated these against total hemibody motor UPDRS scores. The Spearman's correlation coefficient (rho) of mean beta 1 CV vs UPDRS was − 0.44 (p = 0.008) and − 0.59 for mean beta 2 CV (p < 0.001). This procedure was repeated for alpha (7–11 Hz) which demonstrated a weaker correlation with borderline significance (rho = − 0.34, p = 0.044) and for gamma (40–80 Hz), which revealed no correlation (rho = − 0.08, p = 0.65).

The relationship between beta CV and clinical features was not only frequency but also impairment selective. Correlations between beta 1 and beta 2 CV and contralateral rigidity–bradykinesia hemibody scores alone were significant (rho = − 0.45, p = 0.006 and rho = − 0.53, p < 0.001), but there was no relationship between beta 1 and beta 2 CV and contralateral tremor hemibody scores (rho = − 0.26, p = 0.11 and rho = − 0.24, p = 0.16). Correlations were also found to be highly focal. The primary analysis was conducted using STN recordings made at the physiologically defined target level (Yoshida et al., 2010). We therefore repeated the analysis using data recorded from 2 mm above the physiological target level and correlations were absent for both beta 1 and beta 2 (rho = − 0.22, p = 0.2 and rho = − 0.18, p = 0.3).

CV is relatively scale independent, as it is normalised by mean amplitude. To be sure that we were not confounding our predictions of the variance in clinical state with a dependency of the latter on amplitude, we further performed multiple regression analysis using CV and amplitude in the two beta sub-bands. The overall model accounted for 41% of the variance in contralateral hemibody UPDRS scores (r^2^ = 0.41; F(4,31) = 7.060, p < 0.001) and 29% of contralateral rigidity–bradykinesia hemibody UPDRS scores (r^2^ = 0.29; F(4,31) = 4.622, p = 0.005). However, only the CV in the beta 2 band strongly correlated with contralateral hemibody UPDRS scores and was a significant predictor of clinical impairment (standardised *b* coefficient − 0.54, p = 0.009), when accounting for the remaining variables in the regression model.

### On/Off dataset (10 subjects, 17 sides)

We next investigated whether treatment with levodopa increased the CV and did so in proportion to treatment induced improvement in rigidity–bradykinesia. To this end we analysed a second archival dataset that recorded STN LFPs in patients both off and on levodopa, concurrently with clinical assessments. Two recordings were found to not show a distinct peak within the beta range and were therefore excluded from further analysis. The CV estimated for a further recording was identified as a significant outlier and was excluded following examination of residuals (Cook's score 4.6). An ANOVA of amplitude in the remaining 17 sides, with factor medication and beta frequency range (beta 1 or beta 2), revealed an effect of levodopa (F(1,16) = 6.57, p = 0.021) and a trend towards interaction between medication and beta range (F(1,16) = 3.29, p = 0.089) but no main effect of beta frequency range. Post-hoc t-tests showed that there was a significant reduction in beta 1 amplitude (38%; p = 0.03) and a smaller but significant reduction in beta 2 amplitude (21%; p = 0.042). An ANOVA of CV with the same factors revealed no main effects or interactions.

**Fig. 4:**
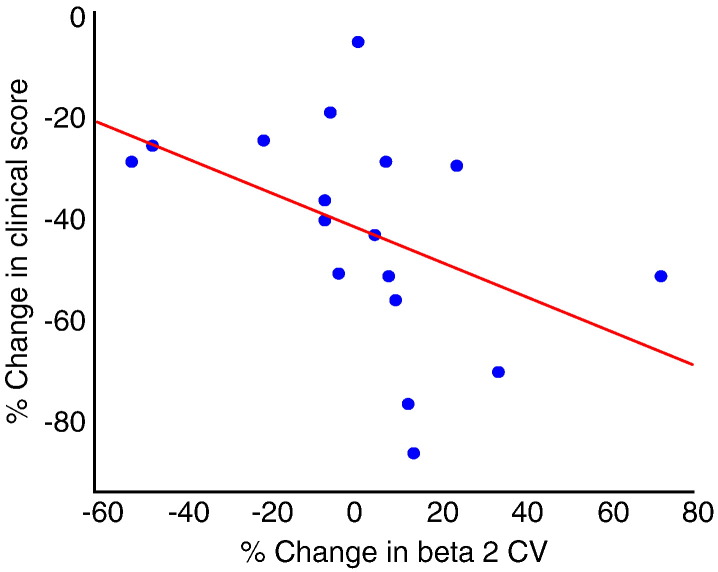
Scatter plot of change in mean beta 2 CV and change in contralateral hemibody rigidity-bradykinesia score in response to levodopa. The patients with the best improvement (more negative % change) in rigidity-bradykinesia have the greater increase in beta 2 CV.

Despite the above, we found a significant negative correlation between the levodopa-induced change in mean beta 2 CV and the change in contralateral rigidity–bradykinesia scores (rho = − 0.66, p = 0.004; Fig. 4). Greater increases in CV upon treatment were associated with greater improvements in rigidity–bradykinesia upon treatment. This was again found to be symptom selective as there was no relationship between beta 2 CV and contralateral tremor hemibody scores (rho = 0.14, p = 0.6). There was also no significant association between change in beta 1 CV and change in clinical state. Note too that the change in CV cannot alone account for improvement in clinical state with medication, as a minority of patients showed modest improvement despite drops in CV after treatment. On two sides, each from different patients, the drop in CV after treatment related to the complete suppression of the beta 2 peak that was formerly present in the off medication state.

Levodopa-induced changes in beta band amplitude are known to correlate with improvements in motor signs. We therefore reprised our previous multiple regression analysis, but using levodopa-induced changes in amplitude, CV and clinical state. The overall model accounted for 44% of the variance in contralateral hemibody rigidity-bradykinesia scores (r^2^ = 0.44, F(4,12) = 4.20 p = 0.024). However, only the change in CV in the beta 2 band was correlated with the change in contralateral hemibody UPDRS (Standardised *b* coefficient − 1.08, p = 0.019), when accounting for the remaining variables in the regression model.

## Discussion

This study demonstrates that the normalised variability (CV) of the beta frequency band component of the STN LFP over time strongly predicts the variance between patients in rigidity–bradykinesia. Specifically, the greater the CV at rest and the greater the increase in CV following levodopa treatment, the lower the bradykinesia–rigidity score at rest and the greater the improvement in rigidity–bradykinesia with treatment. These effects were found to be frequency and impairment specific, as well as focal.

There are several reports that treatment induced suppressions of beta amplitude or power in the STN LFP correlate with treatment induced clinical improvement (Kühn et al., 2006a; Kühn et al., 2009; Ray et al., 2008). However, it has been less clear whether beta amplitude or power in the off medication state correlates with motor impairment. Several studies found no such correlation (Kühn et al., 2006a; Kühn et al., 2009; Ray et al., 2008; Weinberger et al., 2006). In contrast, two studies have reported significant, correlations between beta band power and rigidity-bradykinesia in the off-medication state (rho = 0.428 in (López-Azcárate et al., 2010) and rho = 0.329 in (Özkurt et al., 2011)). Two other studies have reported correlations under similar circumstances using the LZC measure in the beta band (Chen et al., 2010) or LFP phase synchronisation across electrode contacts (Pogosyan et al., 2010). Our study reinforces these findings by demonstrating that the CV of beta 2 band LFP activity, in particular, correlates off medication with rigidity–bradykinesia (rho = − 0.59), and extends them by showing that, like treatment induced changes in beta band amplitude or power, treatment induced changes in CV also correlate with treatment induced changes in bradykinesia and rigidity (rho = − 0.66).

Thus another feature of synchronisation, its temporal variability, may impact on clinical state. Time averaged LFP power/amplitude estimates index the strength of synchronisation, the density and spatial extent of the involved neural pool and the constancy of these features over time. However, power/amplitude estimates cannot infer the relative contributions of these different features and their relative pathophysiological importance remains unclear. Thus, measures reflecting individual features, like the spatial extent of phase synchrony in the beta band (Pogosyan et al., 2010) and the CV of beta band activity may potentially provide complementary, rather than redundant, information about clinical state. This raises the possibility that measures can be combined to strengthen clinical correlations. Özkurt et al. (2011) showed this with beta power and the high frequency oscillation power ratio, and here we demonstrate that combining beta amplitude and beta CV in a multiple regression model accounts for 41% of the variance in contralateral hemibody UPDRS scores off medication. Similarly, combining levodopa-induced change in beta amplitude and beta CV in a multiple regression model accounts for 44% of the variance in change in contralateral hemibody rigidity–bradykinesia scores. This multivariate approach may prove useful in selecting feedback signals that faithfully reflect current motor state and are therefore suitable for closed-loop control of DBS (Rosin et al., 2011). This may be particularly the case with beta amplitude and its CV, as both are computationally simple to calculate, potentially limiting drains on battery power, and correlate over a large spectrum of clinical state as suggested by the correlations evident with rest and treatment induced changes. Nevertheless, it is important to stress that although beta amplitude and CV have been shown to explain a significant portion of the variance in rigidity-bradykinesia across patients, this has yet to be demonstrated within subjects, a necessary pre-requisite for any signal or signal combination to be used in closed loop control.

It should be noted that the OFF drug motor UPDRS scores contrasted with the first intra-operatively recoded LFP data set were assessed a median of 2 months prior to surgery (range 1 day–6 months). This is likely to have, if anything, led to an under-estimation of clinical correlations with CV. Still, other studies demonstrating a correlation between drug-induced change in beta band LFP activity and change in bradykinesia–rigidity have been based on motor examinations that were performed up to a few months before surgery and have shown no difference in the residuals from the correlations between patients examined in this way and those assessed on the same day as electrophysiological recordings (Kühn et al., 2009; Ray et al., 2008). This may be because the changes in UPDRS motor scores over comparable time intervals can be small and insignificant (Olanow et al., 2004).

An interesting aspect of the current study that has thus far not been discussed is the relative focussing of correlations with bradykinesia–rigidity in the beta 2 rather than beta 1 frequency band. This is in accord with the differential reactivities in these two bands under different experimental conditions, including a more pronounced response to treatment with levodopa in the beta 1 than beta 2 range (López-Azcárate et al., 2010; Marceglia et al., 2006, 2007; Priori et al., 2004). Of the two bands, STN activity in the beta 2 band is most synchronised with cortical activity (Hirschmann et al., 2011; Litvak et al., 2011) and may therefore have the greater facility to directly disrupt motor function. However, it has also been suggested that it is not so much the precise frequency of synchronisation in the beta band that may be important, but rather how strong synchronisation is (Kühn et al., 2009). For the moment, the relative contributions of beta 1 and beta 2 frequency bands, if any, to motor impairment remain to be clarified.

Where does the temporal variability of beta activity arise? Could it be an emergent property of stochastic noise in the underlying neural networks, or secondary to deterministic processes? Modelling studies have suggested that beta oscillations in basal ganglia networks are at the boundary of synchronous and non-synchronous regimes in Parkinson's disease, and that small fluctuations in intermittent synchrony can be generated just by moderately increased coupling strength in the basal ganglia circuits due to the lack of dopamine (Park et al., 2011). Dopaminergic therapy suppresses tonic levels of beta (Hammond et al., 2007) and it has recently been suggested that the phasic release of endogenous dopamine in response to salient cues in the internal and external environment causes moment-to-moment fluctuations in beta activity in the basal ganglia (Jenkinson and Brown, 2011). Smaller CVs might then reflect greater attenuation of reactive dopamine release secondary to greater dopaminergic denervation.

Correlation does not necessarily imply causation and whether the behaviour of neuronal populations in the beta band is causally linked to bradykinesia and rigidity or the relationship between the electrophysiological and clinical state is epiphenonal remains to be established (Jenkinson and Brown, 2011; Timmermann and Florin, 2012; Weinberger et al., 2009). Assuming that temporal variability in the degree of synchronisation is causally important, how might this come about? Our data would suggest that it is not simply secondary to increasing levels of background beta synchrony, as the correlation between temporal variability and motor state persisted when we normalised variability by amplitude, and remained significant in a multiple regression model that included amplitude. The implication is that there may be something disadvantageous to basal ganglia function about loss of spontaneous beta reactivity that is relatively independent of the absolute level of beta synchrony. One possibility is that predictive preparation for voluntary action is diminished without phasic modulation of dopamine and beta (Jenkinson and Brown, 2011).
